# Unveiling mechanisms of lung aging in COPD: A promising target for therapeutics development

**DOI:** 10.1016/j.pccm.2024.08.007

**Published:** 2024-09-17

**Authors:** Justine V. Devulder

**Affiliations:** National Heart and Lung Institute, Imperial College London, London SW3 6LY, UK

**Keywords:** Chronic obstructive pulmonary disease, Aging, Cellular senescence, Senescence-associated secretory phenotype, MicroRNAs

## Abstract

Chronic obstructive pulmonary disease (COPD) is a chronic inflammatory lung disease characterized by airflow limitation and changes in airway structures that can lead to chronic bronchitis, small airway diseases, and emphysema. COPD is the 3^rd^ leading cause of death worldwide and despite current research, there are no curative disease treatments for COPD. As the prevalence of COPD is higher in people over 60 years old than in younger age groups, COPD is considered a condition of accelerated lung aging. Natural lung aging is associated with molecular, cellular, and physiological changes that cause alteration in lung structure, in lung function and regeneration, and decreased immune system response that could lead to lung disease like COPD. Mechanisms of accelerated lung aging are complex and composed by increased oxidative stress induced by exposure to cigarette smoke, by chronic inflammatory processes, and increased number of senescent cells within the airways. Cellular senescence is the cessation of cell division after a finite number of proliferation cycles or in response to cell stressors, such as oxidative stress. Senescent cells show activation of the cell cycle regulators p21^CIP1^ (cyclin-dependent kinase inhibitor-1), p16^INK4^ (cyclin-dependent kinase inhibitor-2A), and p53 (cellular tumor antigen p53) that lead to cell cycle arrest. Senescent cells exhibit a change in their phenotype and their metabolic activity, along with the production of proinflammatory proteins collectively known as senescence-associated secretory phenotype (SASP). This review aims to describe recent developments in our understanding of aging mechanisms and how the acceleration of lung aging participates in COPD pathophysiology and comorbidities. Understanding and targeting aging mechanisms may result in the development of new therapeutics that could be effective for COPD and also for other age-related diseases.

## Introduction

Chronic obstructive pulmonary disease (COPD) is a progressive respiratory condition characterized by airflow limitation, persistent inflammation, and irreversible parenchymal lung tissue destruction.^1^ COPD is the 3^rd^ leading cause of death worldwide^2^ and despite many years of research, there are currently no curative treatments for COPD. The acceleration of lung aging has been identified as an essential driver of COPD pathophysiology. The molecular drivers of aging in COPD are multifactorial and they still need to be fully identified. In this review, we provide an overview of the aging biomarkers, of the mechanisms of normal aging in the lungs, and how these mechanisms play a role in COPD pathophysiology.

## Mechanisms of normal aging

Aging is currently defined as “the process of accumulation of consequences of life, such as molecular and cellular damages, that leads to functional decline, chronic diseases, and ultimately mortality”.^3^ Aging not only affects humans, animals, plants, but also unicellular organisms subjected to environmental stresses such as bacteria, protozoa, and fungi.^4^ Aging is a physiological process, however, its mechanisms contribute to the development of diseases,^5^ and has major consequences for the burden of health care.^6^ In Australia, over 80% of patients over 75 years old have multimorbidity,^7^ and in a Mediterranean cohort of people over 85 years, 95% have multimorbidity.^8^ Thus, multidisciplinary studies and new models are critically needed to uncover the mechanisms of aging and age-related diseases.

### Biomarkers of aging

**Fig. 1:**
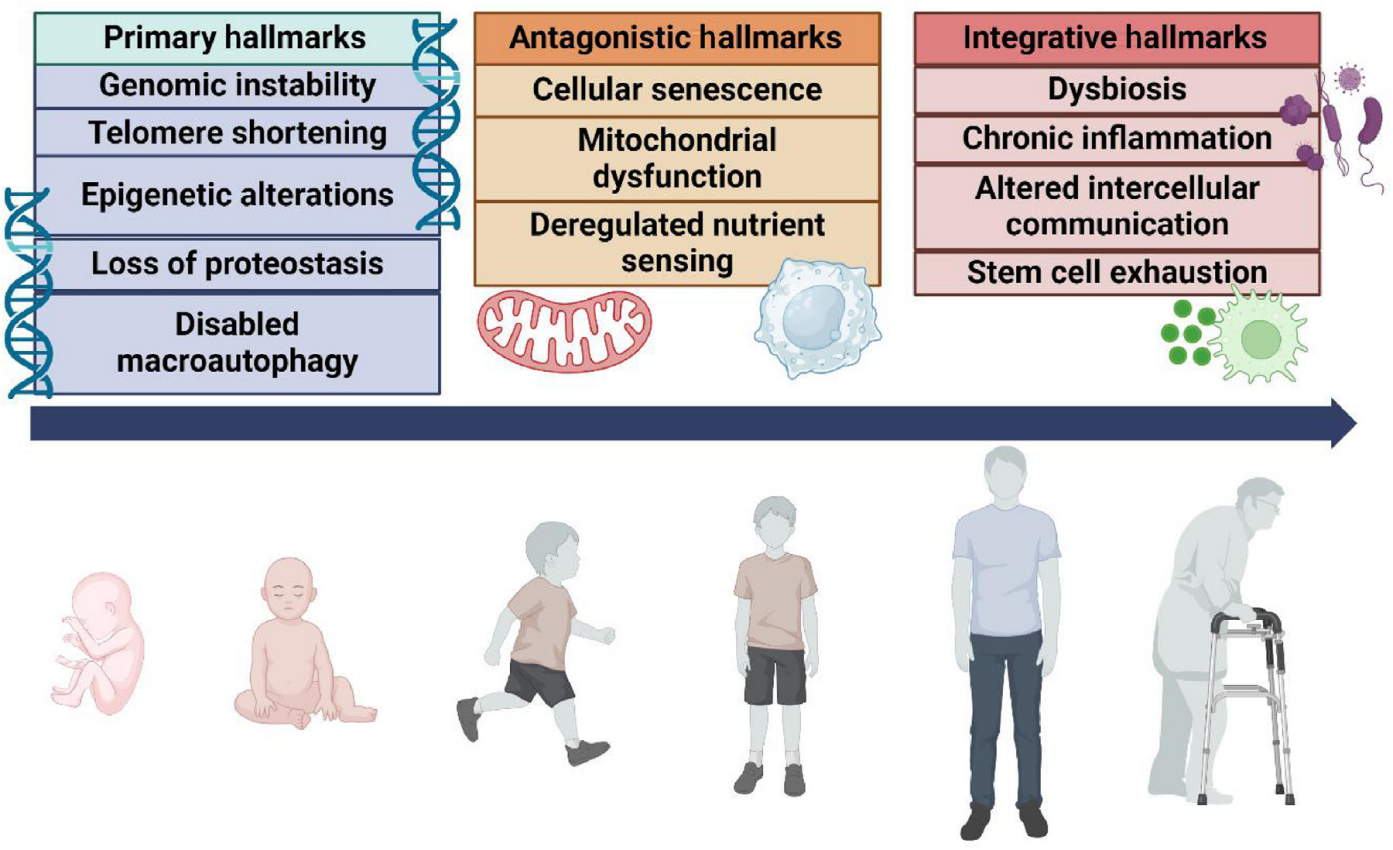
Hallmarks of aging. Twelve hallmarks of aging have been proposed and classified into 3 categories. The primary hallmarks are the primary damages that accumulate within the genome and organelles of the cells. They compile genomic instability, telomere shortening, epigenetic alterations, loss of proteostasis, and disabled macroautophagy. The antagonistic hallmarks reflect the consequences of the damages accumulated within the cells including cellular senescence, mitochondrial dysfunction and deregulated nutrient sensing. Finally, the integrative hallmarks arise when the accumulation of the primary and antagonistic damages cannot be compensated within the organ. They include dysbiosis, chronic inflammation, altered intercellular communication, and stem cell exhaustion.

As the number of people over 80 years of age will quadruple in the next few decades, reliable biomarkers for age-related diseases have become critical tools to target aging mechanisms and, to develop new therapeutics.^9^ Aging mechanisms are highly complex and heterogeneous. Not all individuals age in the same way or at the same rate. These inter-individual differences have led to the definition of chronological age—the individual's age defined by the time elapsed since birth, and biological age—the level of biological changes, such as molecular and cellular damages accumulation.^3^ Numerous studies have attempted to discover biomarkers of aging on different levels: molecular and cellular biomarkers, systemic markers, multi-omics approaches, or non-molecular biomarkers.^10^ In 2023, López-Otin *et al*^11^^,^^12^ have defined 12 hallmarks of aging classified into three categories [Fig. 1]: the primary hallmarks are the primary damages that accumulate within the genome and organelles of the cells, the antagonistic hallmarks that reflect the consequences of the damages accumulated within the cells, and the integrative hallmarks that arise when the accumulation of the primary and antagonistic damages cannot be compensated within the organ.^12^

### Cellular senescence and its consequences in aging

Aging is characterized by the overall decline of cell functions. The genome is instable with increasing levels of mutations, shortening of telomeres, and epigenetic modification. The proteostasis network has its functions depleted, which leads to the accumulation of misfolded proteins.

Telomeres are portions at the end of linear chromosomes that shorten by 50–200 bp at each somatic division.^13^ There is a correlation between age and the relative telomere length across tissue: an analysis of telomere length in 25 tissues collected from 952 donors aged from 20 years to 70 years by the genotype-tissue expression (GTEx) showed telomere length was variable depending on the tissue and was inversely correlated with age in 23 tissues out of 25.^14^ The shortening of telomeres activates the DNA damage response (DDR) which can lead to apoptosis, oncogenic transformation of cells, and to cellular senescence.^15^

Cellular senescence is a stable and terminal state of growth arrest in which cells are unable to proliferate despite optimal growth and mitogenic conditions. Cellular senescence occurs in response to many different triggers, including DNA damage, telomere shortening, oncogene activation, and organelle stress.^16^ Cellular senescence can be beneficial especially during the early stages of development, however, the accumulation of senescent cells within tissues and organs promotes the overall aging of the organism.^17^ Increased number of senescent cells has been reported in older mice and humans. The expression of p21^CIP1^, p16^INK4^, and the activity of β-galactosidase are increased in 120-week mice compared to 15-week mice.^18^ Cellular senescence is initiated and regulated by specific signaling pathways. Telomere attrition or DNA damage activates the DDR and the canonical p53 pathway, whereas cell stress, like oxidative stress, causes cellular senescence via p16^INK4^–Rb pathway. P53 and p16^INK4^ may interact and activate the cyclin-dependent kinase inhibitor p21^CIP1^ which inhibits the formation of the CDK4/6–cyclin D complex, leading to the cell cycle arrest.^19^ A lysosomal enzyme called senescent-associated (SA)-β-galactosidase is increased in senescent cells which is easily detectable by histochemical staining.^17^ Senescent cells release a combination of proinflammatory cytokines, chemokines, proteases, growth factors, and bioactive lipids collectively known as senescence-associated secretory phenotype (SASP). The secretion of SASP is mainly controlled by the activation of p38 mitogen-activated protein kinase (MAPK) and Janus kinase (JAK) that leads to the activation of the nuclear factor κB (NF-κB) pathway.^20^ Other pathways and other upstream factors can also induce cellular senescence and the secretion of SASP. The phosphoinositide-3-kinase (PI3K)-mammalian target of rapamycin (mTOR) pathway can act as a switch where the activation of mTOR causes cellular senescence but inhibition leads to quiescence.^21^ Moreover, the activation of mTOR induces the production of SASP.^22^ SASP release leads to low-grade chronic inflammation called inflammaging.^23^ Immune cells play a crucial role in recognizing and eliminating senescent cells, however, with aging, intercellular communication is altered, and immune cells, in response to SASP, become senescent which leads to a phenomenon called immunosenescence.^24^ Peripheral blood mononuclear cells (PBMC) from people over 60 years exhibit higher activity of β-galactosidase compared to individuals in their 20s, particularly in CD8^+^ T cells among which 64% showed high senescence-associated β-galactosidase, associated with increased expression of p16^INK4^ and telomere damage.^25^

Cellular senescence is characterized by defective autophagy, defined as the catabolic process that enables the degradation and recycling of cellular components, including damaged organelles, pathogens, and protein aggregates.^26^ Autophagy is involved in the recognition and the sequestration of these components into the autophagosome. Mature autophagosomes are transported and fused with lysosomes, which leads to the formation of autolysosomes and the ultimate degradation of the cargo.^26^ Studies suggest that aging is associated with decreased autophagy. The expression of autophagy related 7 (ATG7) and the lipidation of microtubule-associated protein 1A/1B-light chain 3 (LC3) are significantly decreased in the muscle of 26-month-old mice compared to 10-month-old mice. Moreover, this decrease was also found in muscle biopsies of elderly men compared to young adults.^27^

Stem cells have essential roles in tissue development, renewal, and regeneration. Stem cells are tissue-specific, and they remain in a quiescent state within the tissue, for example, in skin, muscle, blood, bone, or brain. The hallmarks of aging, genome instability, epigenetic modification, increased cellular senescence, and subsequent low-grade inflammation, also affect stem cells. As a result, stem cells undergo a progressive decline in homeostatic and regenerative capacities.^28^ Transcriptomic analysis of muscle in older mice has shown that the increased number of senescent cells create a microenvironment, a niche that is inflamed and induces the arrest of stem cell proliferation and regeneration. This interaction between senescence and stem cells could be a therapeutic target as the inhibition of SASP production accelerates regeneration by stem cells in old and young mice.^29^

## Normal aging in the lungs

**Fig. 2:**
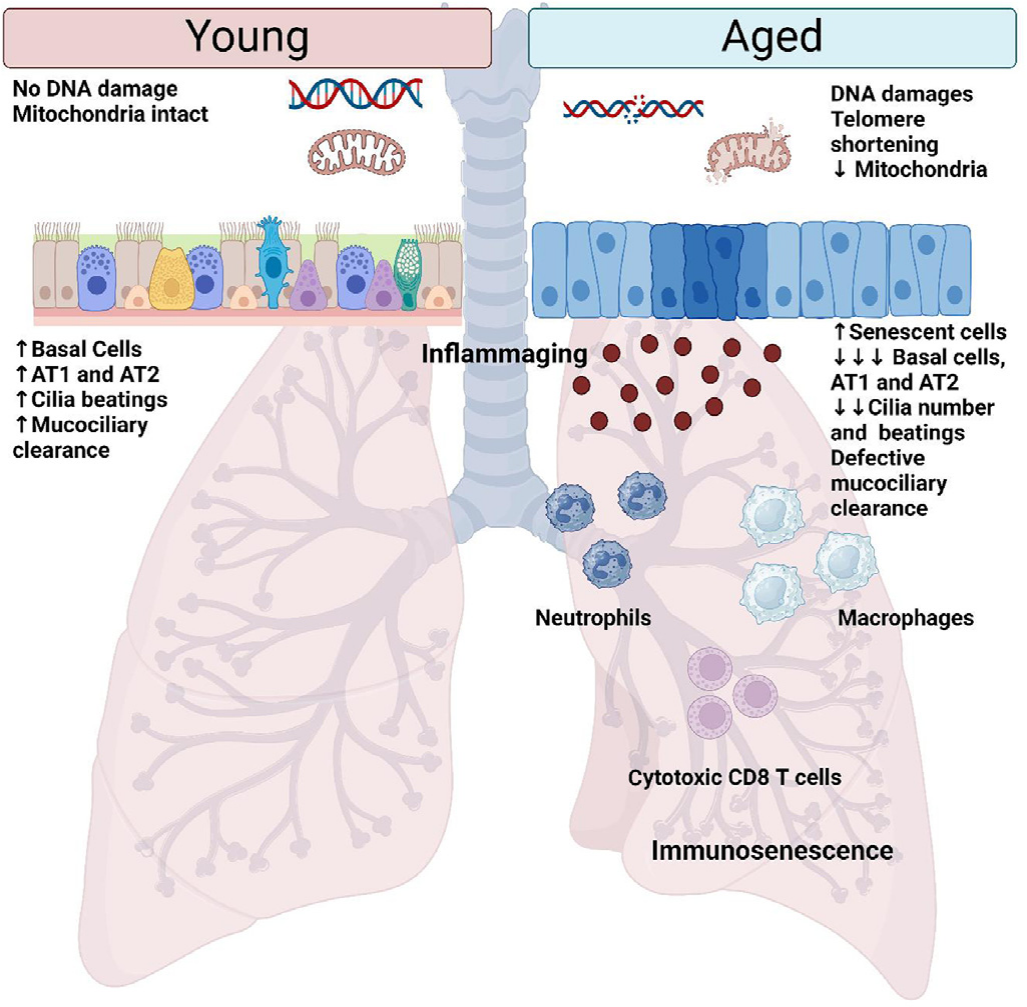
Normal aging of the lungs. Normal aging of the lungs is characterized by the accumulation of DNA damages and oxidative stress that leads to the accumulation of senescent cells, particularly within the small airways. As a result, the epithelium is defective and the number of basal cells, AT1 and AT2 cells diminished. Moreover, the defective mucociliary clearance due to the decreased number of cilia and their defective beating, leads to an increased susceptibility of the elderly to infection. Airway immune response is dysfunctional with aging and is characterized by the accumulation of inflammatory cells such as macrophages, neutrophils, and cytotoxic T cells, within the lungs. These immune cells have decreased functions and may present a senescent phenotype. Aged lungs are also characterized by the production of pro-inflammatory factors, by senescent cells and immune cells, which induce a low-grade inflammation of the lungs called inflammaging. AT1: Alveolar type 1 cell; AT2: Alveolar type 2 cell.

In healthy aging, the respiratory tract undergoes structural, cellular, and immunological changes [Fig. 2]. As a result, the remodeling of the lungs of the elderly leads to the decline of their pulmonary function.^30^ Lung functions measured with the forced expiratory volume in 1 s (FEV_1_), forced vital capacity (FVC), and peak expiratory flow rate (PEFR) reach their maximum capacity around 25 years old, and progressively decline with age (rates of FEV_1_ decline ranged from 17.7 mL/year to 46.4 mL/year).^31^

### Structural changes in the lungs

The respiratory system is composed of the lungs, the thoracic cage, and the diaphragm, and each of these is modified by aging. Lung compliance decreases with age, which is due to a general loss of the elasticity of the respiratory system.^32^ Indeed, the size of the thoracic cavity and rib spaces can decrease because of bones weakening, and there is an association between the decreased pulmonary function and osteoporosis.^33^ Respiratory function is also impaired because of muscle atrophy and weakness associated with aging. Intercostal, inspiratory, and expiratory muscles, and the diaphragm lose their contractile capacity, their mass, and their strength with age.^34^^,^^35^ This contributes to reduce cough strength and thus the ability of elderly lungs to clear mucus. Multiple studies have shown that mucociliary transport is slowed in the upper and lower airways of elderly people. In a cohort of 46 healthy subjects aged 19–81 years old that inhaled labeled Teflon particles, the small airway clearance was negatively correlated with age, thus older people present a delayed clearance of particles in the small airways.^36^ In nasal epithelial cells isolated from people between 11 years and 90 years of age, a negative correlation between the ciliary beat frequency and age has been confirmed.^37^ Finally, even if the number of alveoli does not seem to be modified by aging, the enlargement and decreased elasticity of alveoli associated with the destruction of lung parenchyma can cause senile emphysema.^38^

### Cellular changes associated with aging in the lungs

Over the course of a lifetime, the respiratory tract is continuously exposed to environmental injuries. Consequently, mechanisms of repair are of utmost importance and their dysfunction can lead to fibrosis and remodeling of the lungs. Aging is associated with decreased epithelial barrier function. *In vitro* culture of bronchial epithelial cells from subjects aged over 45 showed a decreased transepithelial resistance with increasing age.^39^ Single-cell analysis has shown that the pulmonary epithelium undergoes dynamic changes during aging. The number of basal cells decreases with age as well as the ratio between epithelial cells and basal cells. Moreover, the function of basal cells, alveolar type (AT)1, and AT2 cells diminishes with aging suggesting the impairment of airway regeneration and repair. In a mouse model, deficiency of the telomerase reverse transcriptase leads to the increased concentration of pro-inflammatory cytokines in the lungs associated with AT2 cell senescence.^40^ Moreover, AT2 cells isolated from human lungs showed a diminished proliferation rate, elevated cellular senescence, loss of epithelial markers, increased expression of activating protein-1 (AP-1), and chemokine genes in people over 60 years compared to those of 20 years.^41^ Composition of extracellular matrix (ECM) may also change with aging. ECM from the lungs of old mice (20–24 months) presents a decreased amount of laminin and increased expression of collagen I and fibronectine compared to 4-month-old to 6-month-old mice. Thus, the aged ECM becomes stiffer which leads to biomechanical alterations.^42^ In aged lungs, endothelial cells function declines due to cellular senescence and a decreased production of endothelial growth factor that impacts angiogenesis.^43^ The increased cellular senescence of the endothelium has been attributed to an increased vulnerability to oxidative stress, a decline in the number of stem cells leading to defective repair, and impaired nitric oxide signaling.^44^

### Immunological changes associated with aging in the lungs

#### Innate immunity

Pathogen recognition and elimination is impaired with aging, leading to more acute and chronic pathology in the elderly.^45^ Alveolar macrophages are key effectors in the immune clearance of microbes in the lungs. Alveolar macrophages recognition of pathogen-associated molecular patterns (PAMPs) and damage-associated molecular patterns (DAMPs) is key to initiate immune response. In a mouse model infected by *Streptococcus pneumoniae* (*S. pneumoniae*), alveolar macrophages isolated from old mice (21 months old) present a defective activation of Toll-like receptor 2 (TLR2) signaling leading to a delayed pro-inflammatory cytokines responses associated with enhanced susceptibility to pneumonia compared to young mice (4 months old).^46^ Moreover, alveolar macrophages change intrinsically with aging, and exhibit a decrease in cell numbers and altered gene expression, which leads to a defective phagocytosis and high production of pro-inflammatory cytokines and chemokines.^47^^,^^48^ In a mouse model of infection with *S. pneumoniae*, the number of mitochondria decreased in alveolar macrophages isolated from older mice (19 months old) compared to young mice (2 months old). This was associated with decreased adenosine 5′-triphosphate (ATP) production, diminished antioxidant response and increased production of reactive oxygen species (ROS), and was reversed by the administration of pirfenidone, an anti-fibrotic drug with antioxidant properties, suggesting that oxidative stress may drive the aging of macrophages.^49^ Macrophages work in conjunction with neutrophils to contain and clear infections. Neutrophils are quickly recruited at the sites of infection to carry out their microbicidal activity by phagocytosis, degranulation of antimicrobial proteins, and the release of neutrophil extracellular trap.^45^ With aging, the recruitment of neutrophils is decreased despite equivalent levels of chemokines chemokine (C-X-C motif) ligand 2 (CXCL2) and C-C motif chemokine ligand 2 (CCL2) at the site of infections, suggesting that neutrophil chemotaxis is dysregulated in aging.^50^ Thus, recruitment and function of neutrophils are impaired in elderly patients, and this may be due to diminished activity of nicotinamide adenine dinucleotide phosphate (NADPH) oxidase and myeloperoxidase (MPO) that could lead to oxidative stress.^51^

#### Adaptive immunity

Similar to the innate immune response, the adaptive response is also considerably modified by aging mechanisms. During aging, hematopoietic stem cells in the bone marrow show reduced self-renewal, leading to the decreased generation of B and T lymphocytes.^52^ Other studies have reported modification of the phenotype of hematopoietic stem cells with aging with modification of their frequencies, and increased expression of markers of myeloid lineage.^53^ With advancing age, the naïve B cell pool diminishes while the memory B cell pool expands. Changes in pro-B, pre-B, and immature B cells are associated with a decrease in repertoire diversity leading to low antibody affinity and impaired class switching. B cells are thus more prone to produce auto-antibodies.^54^ In the same way, the pool of naïve T cells during aging is in favor of the accumulation of differentiated and less proliferative T memory cells and effector memory T cells re-expressing CD45RA (TEMRA).^55^ The single-cell analysis of T cells isolated from young and old mice showed that CD4^+^ T cells undergo a gradual reorganization with accumulation of regulatory, exhausted, and cytotoxic phenotype with aging.^56^ In bronchoalveolar lavage (BAL) fluid, the ratio of CD4^+^ to CD8^+^ T lymphocytes increases with age, confirming the expansion of memory T cells.^43^ A longitudinal study of T-cell receptors (TCR) repertoire of blood CD4^+^ and CD8^+^ T cells showed an alteration of TCR repertoire with age. The diminution of TCR repertoire was subset specific with the greatest reduction observed in naïve CD8^+^ T cells.^57^ Thus, the adaptive immune system in the elderly has a decreased capacity to recognize microbial agents, which leads to increased susceptibility to infectious stimuli and prolonged and more severe disease.

## Lung aging and COPD

COPD is a complex and multifactorial condition that develops following chronic exposure to hazardous compounds. It is the third leading cause of death globally. In 2017, the number of people living with chronic respiratory disease was estimated to be 544.9 million, and approximately 55% of cases can be attributed to COPD.^58^ The pathophysiology of COPD is driven by cascade of events that include aberrant immune responses and cellular mechanisms that lead to persistent inflammation, fibrosis, and lung damage.^59^ These molecular and cellular events are similar to those characterized in aging and the acceleration of lung aging is an essential feature of COPD pathophysiology.

### Age is a risk factor for developing COPD

The first risk factor identified for COPD is cigarette smoking. According to the Global Initiative for Chronic Obstructive Pulmonary Disease (GOLD), half of smokers will eventually develop COPD.^60^^,^^61^ However, between 25% and 45% of COPD patients have never smoked and therefore other risk factors have been identified: occupational dusts, vapors and fumes, biomass fuels, outdoor pollutants, and age.^62^ Diagnosis of COPD is made around 60 years old, therefore COPD affects preferentially elderly patients and has dramatic impacts on their health and quality of life.^63^ Between 40 and 59 years of age, the prevalence of COPD is 9.2% and it increases to 22.6% in people between 60 and 79 years old.^64^ It was unclear if aging was implicated in COPD pathophysiology. Two biomarkers of biological aging, dehydroepiandrosterone (DHEA) and growth hormone (GH), were measured in the blood of healthy subject and COPD patients. Both hormones were negatively correlated with age, and the levels of DHEA and GH were significantly decreased in the blood of COPD patients compared to aged-matched healthy individuals. The biological age of COPD patients was estimated to be 13–24 years older than healthy donors. Thus, premature aging, particularly of the lungs, is key in COPD pathology.^65^

### Hallmarks of lung aging in COPD

#### Oxidative stress in COPD lungs

Oxidative stress is the accumulation of free radicals due to the overproduction of ROS that cannot be processed gradually by the cells.^66^ During normal aging, chronic oxidative stress arises in cells due to the overproduction of ROS, along with a decline in ATP and antioxidants production. This participates in aging through telomere shortening, genetic instabilities, epigenetic modification, alteration of mitochondria functions, and cellular senescence.^67^ The imbalance between the production of ROS and antioxidants has been characterized in COPD patients. In exhaled breath condensate of COPD patients, increased levels of ethane, H_2_O_2_, malondialdehyde (MDA), 4-hydroxynonenal (HNE), or 8-isoprostane have been measured compared to smokers without COPD and healthy non-smokers,^68^, ^69^, ^70^, ^71^ whereas the levels of antioxidants glutathione (GSH), superoxide dismutase (SOD), and GSH peroxidase (GSH-PX) are reduced.^72^ In COPD patients, oxidative stress is generated by exogenous products, like cigarette smoke, but also by endogenous production from increased numbers and activation of inflammatory cells, such as leading to a sustained oxidative stress even after smoking cessation.^73^ In COPD lungs, oxidative species are mainly produced by inflammatory cells. Indeed, the immune response is altered in COPD patients and leads to the recruitment of pro-inflammatory cells within the lungs, leading to the chronic inflammation of the airways. Among these cells, macrophages and neutrophils are activated in COPD and secrete high levels of superoxide (O_2_^−^) and hydrogen peroxide (H_2_O_2_).^74^ Moreover, the expression of nuclear factor erythroid 2-related factor (Nrf2), a master transcription factor that has an antioxidant activity, is decreased in alveolar macrophages from COPD patients,^75^ further suggesting a decline in antioxidant activity of immune cells in COPD.

The chronic imbalance between oxidants and antioxidants leads to cellular damage in the airways and the alveolar spaces of COPD patients. Bronchial epithelial cells, AT2 cells, endothelial cells, macrophages, and fibroblasts exhibit multiple oxidative stress damages, including lipid peroxidation, protein oxidation, DNA damages, telomeres shortenings, inhibition of DNA methylation, and mitochondrial dysfunction, all of which have been identified as aging mechanisms.^76^ Oxidative stress participates in glucocorticosteroid resistance of COPD patients by driving the phosphorylation of glucocorticoid receptor (GR) as well as the loss of histone deacetylase-2 (HDAC-2).^77^ Overproduction of ROS participates in chronic bronchitis and the development of mucus plugging by stimulating the production of mucin genes like *MUC5b* and *MUC5ac*.^78^^,^^79^ Production of pro-inflammatory factors is promoted by oxidative stress through the NF-κB and the dissociation of the inhibitor of NF-κB (IκB)/NF-κB complex in airway epithelial cells and macrophages of COPD patients.^80^^,^^81^ Oxidative stress influences the proteostasis network, particularly through the endoplasmic reticulum (ER)-stress. ER is the largest organelle in the cell and is a major site of protein synthesis and transport.^82^ The consequence of ER stress is the misfolding of secretory proteins which induce the unfolded protein response (UPR) controlled by three pathways: ER-resident protein kinase RNA-like ER kinase (PERK), the endoribonuclease inositol requiring enzyme 1 (IRE1α), and the transcription factor activating transcription factor 6 (ATF6).^83^ In human bronchial epithelial cells from COPD patients, sustained oxidative stress induces the upregulation of UPR markers that can be abrogated when cells are incubated with an antioxidant.^84^ Moreover, ER stress and autophagy are two important cell processes that are closely related. The activation of PERK promotes the formation of the autophagosome through the Atg5–Atg12–Atg16 complex.^85^ Lots of studies have shown that autophagy is impaired in COPD, particularly in response to cigarette smoke and oxidative stress.^86^ However, the interaction between ER stress, autophagy, and COPD is not fully characterized and needs further investigations.

#### Inflammaging in COPD

COPD is characterized by an abnormal chronic inflammatory response of the lungs due to the repeated and progressive activation of immune cells by cigarette smoke and oxidative stress. This leads to a pulmonary and systemic low-grade inflammation similar to the inflammation characterized in normal aging. As such, aged mice exposed to cigarette smoke exhibit more airway remodeling, emphysema, and decreased lung function than young mice, suggesting that inflammaging increases the susceptibility to cigarette smoke.^87^

The innate and adaptive immune responses are impaired in COPD and participate in the development of chronic inflammation of the airways. Airway epithelial cells are key players in the development of inflammaging in COPD as they serve as a molecular and physical barrier for particulate matter and microbes. Repeated exposure to oxidative stress and cigarette smoke is associated with decreased epithelial barrier function, abnormalities in cilia structure and function and reduced production of antimicrobial and anti-inflammatory proteins.^88^ The initial site of these pathological changes in COPD is the small airway epithelial cells where inhaled irritants induce a global reprogramming of small airway epithelial cells toward a phenotype similar to that observed in proximal airways.^89^ As the disease progresses, this reprogramming leads to the gradual destruction of the small airways.^90^

Alveolar macrophages are key player in COPD pathophysiology because of their capacity to orchestrate inflammatory response to inhaled irritants. The number of macrophages is increased by approximately 20-fold in COPD lungs,^91^^,^^92^ where they participate in chronic inflammation of COPD airways and emphysema by producing increasing levels of matrix-metalloproteases (MMP) 2 and MMP9.^93^ The metabolism of alveolar macrophages is modified in COPD: monocytes-derived macrophages isolated from COPD patients showed decreased mitochondrial membrane potential and increased production of ROS that led to defective phagocytosis and may predispose COPD patients to chronic exacerbation particularly in response to bacteria such as *Haemophilus influenzae* and *S. pneumoniae*.^94^^,^^95^ Moreover, pulmonary macrophages regulate the inflammatory response by recruiting innate and adaptive immune cells. In response to oxidative stress, macrophages produce transforming growth factor-β (TGF-β) and C-X-C chemokine ligand 8 (CXCL8), inducing the recruitment of neutrophils in COPD airways.^96^ Efferocytosis is impaired in COPD, leading to an increased number of neutrophils and eosinophils in the airways, correlated with the severity and the frequency of COPD exacerbations.^97^ Production by macrophages of CXCL9, CXCL10, and CXCL11 induces the recruitment of CD4^+^ and CD8^+^ T cells in the lungs of COPD patients. CD4^+^ and CD8^+^ T cells produce perforin and granzyme B that drive the apoptosis of alveolar cells contributing to emphysema.^98^ Altogether, numerous studies have confirmed that immune cell function is impaired in COPD and leads to a low-grade inflammation similar to the inflammatory response described in aging. A study from Maté *et al*^99^ has shown that the function of leukocytes was defective in COPD patients and that they appear to age at a faster rate than in healthy age-matched individuals. However, features of aging in immune cells, like immunosenescence, and mechanisms of aging are still unknown and need to be characterized.

#### Cellular senescence in COPD

Cellular senescence is now considered a major driver of COPD pathophysiology. Higher proportions of senescent AT2 cells, endothelial cells, and smooth muscle cells are detected on lung sections of COPD patients.^100^ Compared to age-matched healthy individuals, small airway fibroblasts and small airway epithelial cells show increased p21^CIP1^ and p16^INK4^ expression along with an increased senescence-associated β-galactosidase staining.^101^^,^^102^

Cellular senescence of the small airway epithelium has been well-characterized and identified as a driving mechanism in COPD pathogenesis.^102^ Cellular senescence is not restricted to epithelial cells and senescent airway structural cells participate in COPD pathophysiology and lead to lung destruction and emphysema.^100^ Dysfunction of the endothelium contributes to COPD pathology and severity as well as cardiovascular disease, a common comorbidity that coexists with COPD.^103^ Endothelial colony-forming cells (ECFC) isolated from healthy smokers and COPD patients express higher levels of senescence markers, p21^CIP1^, p16^INK4^, SA-β-galactosidase and increased markers of DDR, gamma histone 2AX (γH2AX), and tumor protein P53 binding protein 1 (53BP1) compared to ECFC from healthy non-smokers.^104^ Thus, senescent ECFC display impaired angiogenic abilities and participate in the altered pulmonary vasculature observed in COPD. Fibroblasts are key contributors in the development of small airway disease early in COPD pathogenesis. Parenchymal-derived fibroblasts showed altered functions and senescence features in COPD patients with severe emphysema.^105^ COPD small airway fibroblasts display increased p21^CIP1^ and p16^INK4^ expression along with SA-β-galactosidase activity, and mitochondrial dysfunction.^101^ This phenotype may be associated with fibrotic properties which may participate in small airway disease progression in COPD. Airway smooth muscle cells (ASMC) play a central role in the pathogenesis of COPD as they are crucial components of the airway for their contractile function and contribution to the production of inflammatory mediators, proteases, and growth factors.^106^ Airway remodeling characterized in COPD involves airway smooth muscle thickening caused by ASMC hypertrophy and/or hyperplasia.^107^ Impairment of ASMC metabolism has been shown in COPD, particularly the accumulation of lactate, glutamine, fatty acid, and amino acids compared to healthy ASMC.^108^ These modifications are associated with the alteration of intracellular Ca^2+^ signaling, also seen in healthy-aged ASMC.^109^ Moreover, pulmonary-artery smooth muscle cells isolated from COPD patients expressed higher levels of p16^INK4^, associated with the increased staining of β-galactosidase and decreased proliferation rate.^110^ Thus, ASMC senescence could participate in COPD and its comorbidities pathophysiology *via* airways and pulmonary vessel remodeling.

**Fig. 3:**
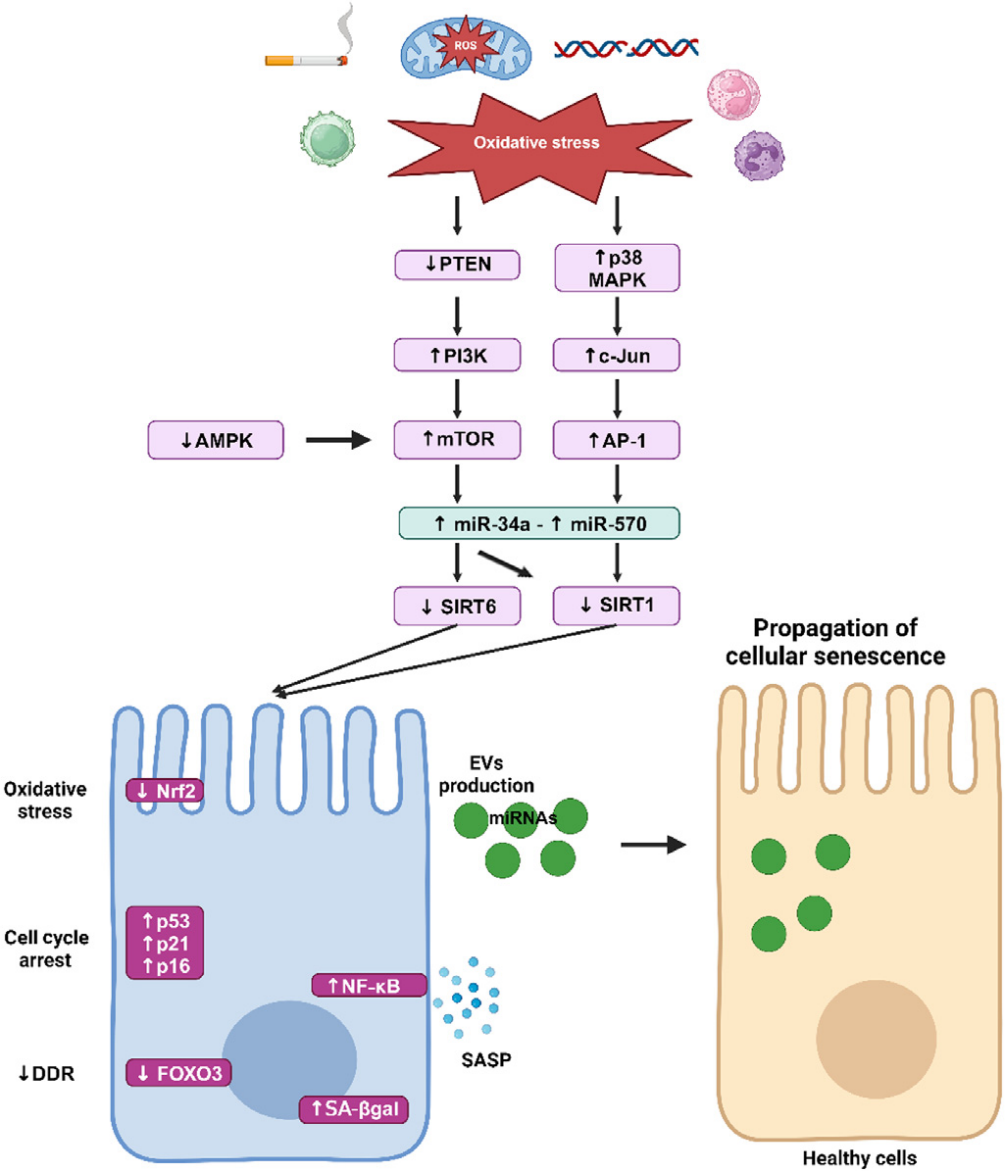
Cellular senescence pathway in COPD. Oxidative stress inhibits PTEN, leading to the activation of PI3K and consequently to the activation of mTOR. Decreased activation of AMPK also increases mTOR activity which leads to the upregulation of microRNA (miR)-34a. Activation of p38 MAPK leads to the upregulation of c-Jun and AP1, which increases miR-570. miR-34a targets SIRT1 and SIRT6 and miR-570 targets SIRT1 but not SIRT6. Decreased SIRT1 and SIRT6 play a key role in the induction of cellular senescence through SASP production via activation of NF-κB and through the increased expression of p53, p21^CIP1^, and p16^INK4^. SIRT1 is involved in the diminution of DDR by inhibiting FOXO3 and participates in mitochondria dysfunction by inhibiting autophagy and PGC1α. Downregulation of SIRT6 inhibits the antioxidant Nrf2 which leads to chronic oxidative stress. Senescent cells produce a large number of EVs that contain miRNAs and induce cellular senescence phenotype to healthy cells, thus participating in the propagation of aging within the lungs. AMPK: AMP-activated protein kinase; AP-1: Activating protein-1; COPD: Chronic obstructive pulmonary disease; DDR: DNA damage response; EVs: Extracellular vesicles; FOXO3: Forkhead box O3; miRNAs: MicroRNAs; MAPK: Mitogen-activated protein kinase; mTOR: Mammalian target of rapamycin; NF-κB: Nuclear factor-κB; Nrf2: Nuclear factor erythroid 2-related factor; PTEN: Phosphatase and tensin homolog from chromosome 10; PGC1α: Peroxisome proliferator-activated receptor-γ coactivator 1-α; PI3K: Phosphoinositide 3-kinase; ROS: Reactive oxygen species; SA-βgal: Senescent-associated β-galactosidase; SASP: Senescence-associated secretory phenotype; SIRT1: Sirtuin 1; SIRT6: Sirtuin 6.

Oxidative stress is an essential inducer of cellular senescence in COPD as it directly activates the p53–p16^INK4a^ pathway as well as damaging the DNA, leading to the activation of p21^CIP1^ [Fig. 3]. Reduced telomere length has been demonstrated in circulating leukocytes of COPD patients. In lung tissues of COPD patients, an increased number of DNA double-strands breaks, γH2AX were detected in AT1, AT2, and endothelial cells and were associated with the activation of p16^INK4^ and the NF-κB pathway.^111^ Failure to repair DNA damage may be related to COPD severity. A DNA repair marker, Ku86, is decreased in parenchymal lung tissue and small airway of COPD patients.^112^ As such, polymorphisms of DNA repair genes have been described in COPD patients and correlated with DNA damage and disease progression.^113^ Moreover, oxidative stress leads to the upregulation of peroxisome proliferator-activated receptor-γ coactivator 1-α (PGC1α) and decreased autophagy in COPD airway epithelial cells.^102^

One of the key players in the regulation of cellular senescence in COPD is the anti-aging molecules sirtuins (SIRT). The SIRT family are nicotinamide adenine dinucleotide (NAD)+ dependent histone deacetylases that are highly conserved across species and comprised of 7 members.^114^ SIRT play a vital role in sustaining genome integrity by maintaining normal chromatin condensation rate, DDR and repair, and modulating oxidative stress and cell metabolism.^115^ Among SIRT members, SIRT1 and SIRT6 have been linked to cellular senescence and aging because of their capacity to regulate inflammatory responses, oxidative stress, and autophagy.^116^ SIRT1 and SIRT6 protect cells from senescence by deacetylating the transcription factor forkhead box O3 (FOXO3) and by inhibiting the PI3K–mTOR pathway.^117^^,^^118^ A reduction of SIRT1 expression has been described in serum, peripheral lungs, airway epithelial cells, and circulating PBMC of COPD patients.^119^, ^120^, ^121^ This reduction of SIRT expression is induced by oxidative stress through the inhibition of the phosphatase and tensin homolog from chromosome 10 (PTEN), which leads to the activation of PI3K–mTOR pathway.^122^ SIRT1 and 6 are downregulated by microRNAs (miRNAs) in COPD.^121^^,^^123^ miRNAs are short, regulatory RNAs that act as post-transcriptional repressor of gene expression.^124^ Dysregulation of miRNAs expression has been highlighted in COPD, as a response to cigarette smoke injury. Among them, upregulation of miR-15b, miR-22, miR-638, and miR-494 and downregulation of miR-24, miR-126, and miR-149 have been described in COPD lung tissues, fibroblasts, and airway epithelial cells.^125^ In a mouse model of emphysema induced by cigarette smoke and in human lung tissues from COPD patients, elevated levels of miR-125a have been detected and were negatively correlated with lung function. Moreover, miR-125a induced cellular senescence by inhibiting a transcription factor called Sp1, which leads to the downregulation of SIRT1.^126^ In small airway epithelial cells from COPD patients, miR-34a and miR-570 are upregulated and their targets SIRT1 and SIRT6 are subsequently downregulated. The activation of the PI3K pathway by oxidative stress leads to the upregulation of miR-34a whereas the activation of p38 MAPK and c-Jun N-terminal kinase signaling leads to the upregulation of miR-570. Most importantly, transfection of COPD small airway epithelial cells with an antagomiR against miR-34a or miR-570 not only restores expression of SIRT1 and SIRT6, but also reduces markers of cellular senescence (p16^INK4^, p21^CIP1^, SASP production, and SA-β-galactosidase).^121^^,^^123^ miRNAs are mainly located in the cytoplasm where they exert their biological function. However, recent studies have localized them in different cell compartments, including their secretion outside the cell in extracellular vesicles (EVs).^125^ EVs are lipid bilayer vesicles secreted by cells and contain a specific cargo composed of proteins, lipids, DNA, RNA, or miRNAs. By carrying and transporting this cargo to other cells, EVs trigger a molecular and/or phenotypic response in recipient cells.^127^ Modification of EVs cargo has been shown in COPD: EVs isolated from plasma of COPD patients contain higher levels of miR-22, miR-99a, miR-151a, miR-320b, and miR-320d compared to EVs isolated from smokers and non-smokers without COPD. Gene set enrichment analysis revealed that the target of these miRNAs plays crucial roles in cytokine signaling and in tissue remodeling,^128^ however, their function in COPD pathophysiology needs further study. Senescent cells produce more EVs and their cargo can be enriched with miR-34a, miR-23b, or miR-494, which can induce cellular senescence.^129^ Thus, EVs can participate in the propagation of cellular senescence within the lungs, which may account for disease severity and comorbidities.

## Conclusions

Considerable progress in understanding the role of accelerated lung aging in COPD has been made during the last decade. Aging is an inexorable process. However, for the best or the worst, people aged differently, suggesting that genetic, epigenetic, and environmental factors interact and contribute to healthy aging or to the development of age-related disease, like COPD. The characterization of biomarkers of aging and a more accurate definition of the biological age would help differentiate healthy from unhealthy aging and will allow the early detection of COPD. Moreover, the current studies mainly fail to integrate data from different organs to allow an early detection of age-related disease in an individual. COPD frequently coexists with other diseases and comorbidities that have significant health and economic consequences.^130^ A recent study by Tian *et al*^131^ performed a multimodal analysis to measure the biological age of seven organs and the brain and showed that lung aging strongly influenced the aging of the cardiovascular system. Thus, the future perspective of COPD is to further understand the pathophysiology of COPD and its comorbidities and to understand how organs communicate and influence each other's aging markers.

The identification of aging biomarkers and signaling pathways involved in COPD has led to new therapeutic targets. Among them, the use of senotherapeutics has become an interesting approach. Senomorphics are agents that block senescence pathways and the production of SASP, including telomerase activators, sirtuin activators, mTOR inhibitors, antioxidants, anti-inflammatory agents, autophagy and proteasome activators. Treatment of lung cells from COPD patients with rapamycin, an mTOR inhibitor, prevents cellular senescence and inhibits the production of SASP. However, SASP factors are not only deleterious, they can have a role in tissue homeostasis, thus senomorphics may lead to serious side effects. The elimination of senescent cells may stop disease progression and favor lung regeneration. Clearance of senescent cells in aged mice attenuated the age-related deterioration of several organs and expanded lifespan by 17% to 42%.^132^^,^^133^ Senolytics are small molecules that eliminate senescent cells by inducing apoptosis. Seven classes of senolytics have been described: natural compounds, kinase inhibitors, B-cell lymphoma-2 (Bcl-2) family inhibitors/Bcl-2 homology 3 (BH3) mimetics, inhibitors of mouse double minute 2 homolog (MDM2)/p53 interactions, heat-shock protein 90 (Hsp90) inhibitors, p53 binding inhibitors, and HDAC inhibitors.^134^ Navitoclax (ABT-263) targets the antiapoptotic proteins Bd-2, B-cell lymphoma-extra large (BCL-X_L)_, and BCL-w and reduces the viability of senescent human umbilical vein endothelial cells (HUVECs), IMR90 human lung fibroblasts, and murine embryonic fibroblasts.^135^ Studies in mice have shown beneficial effect on aged mice, notably their brain function, however, potential harmful effects have also been described on osteoprogenitors.^136^^,^^137^ These novel therapies may have major effects on COPD and other chronic age-related diseases in the future. However, further studies are needed to understand the effect of this therapy on senescent cells and on the regeneration of cells and tissues.

## Declaration of competing interest

None.
